# Improving the Performances of Random Copolymer Based Organic Solar Cells by Adjusting the Film Features of Active Layers Using Mixed Solvents

**DOI:** 10.3390/polym8010004

**Published:** 2015-12-29

**Authors:** Xiangwei Zhu, Kun Lu, Benzheng Xia, Jin Fang, Yifan Zhao, Tianyi Zhao, Zhixiang Wei, Lei Jiang

**Affiliations:** 1 National Center for Nanoscience and Technology, Beijing 100190, China; zhuxw@nanoctr.cn (X.Z.); lvk@nanoctr.cn (K.L.); xiabz@nanoctr.cn (B.X.); fangj@nanoctr.cn (J.F.); zhaoyifan@nanoctr.cn (Y.Z.); 2 Key Laboratory of Bio-Inspired Smart Interfacial Science and Technology of Ministry of Education, School of Chemistry and Environment, Beihang University, Beijing 100191, China

**Keywords:** random copolymer, photovoltaic, mixed solvents, film feature

## Abstract

A novel random copolymer based on donor–acceptor type polymers containing benzodithiophene and dithienosilole as donors and benzothiazole and diketopyrrolopyrrole as acceptors was designed and synthesized by Stille copolymerization, and their optical, electrochemical, charge transport, and photovoltaic properties were investigated. This copolymer with high molecular weight exhibited broad and strong absorption covering the spectra range from 500 to 800 nm with absorption maxima at around 750 nm, which would be very conducive to obtaining large short-circuits current densities. Unlike the general approach using single solvent to prepare the active layer film, mixed solvents were introduced to change the film feature and improve the morphology of the active layer, which lead to a significant improvement of the power conversion efficiency. These results indicate that constructing random copolymer with multiple donor and acceptor monomers and choosing proper mixed solvents to change the characteristics of the film is a very promising way for manufacturing organic solar cells with large current density and high power conversion efficiency.

## 1. Introduction

A mixed bulk heterojunction (BHJ) structure, which consists of a conjugated polymer as an electron donor and a fullerene derivative as an electron acceptor, produces high power conversion efficiency (PCE) in polymer solar cells (PSCs) [1]. Alternating electron donor (D) and acceptor (A) building blocks to construct D–A type polymers is an effective strategy for designing high efficient photovoltaic materials for PSCs [2,3,4]. The properties of D–A copolymers can be fine-tuned by varying the type of electron-donating or withdrawing unit, respectively. In this way, some highly efficient D–A polymers with improved absorption spectra and proper energy levels have emerged in recent years [5,6].

Current D–A copolymer design strategies include: (1) synthesizing novel donor [7] and acceptor units [8]; (2) introducing π-spacers to construct D-π-A polymers [9]; (3) optimizing the alkyl side chains to balance the crystallinity and solubility [10]; and (4) introducing substituents, for example fluorine atoms, to tune the energy levels [11]. In addition to these methods, introducing many different units into the polymer backbone to construct the copolymer is also a promising approach, since they can exhibit the synergetic effects of each unit by tuning the composition ratio, thus exhibiting red-shifted absorption spectra, proper highest occupied molecular orbital (HOMO) energy levels, higher hole mobility, and greatly improved photovoltaic properties [12]. Such a design strategy creates an intriguing possibility to modify the existing D–A type photovoltaic polymers for better performances. Thus far, there are plenty of examples of significant improvement being achieved by randomly copolymerizing one donor and one acceptor with a third monomer [13,14,15,16].

In addition, other strategies have also been carried out to improve the photovoltaic performances of PSCs by tuning the morphologies of the blend films, such as the selecting different preparation solvent, introducing high boil point additive, using mixed solvents and annealing the active layer [17]. Among them, using mixed solvents is a very maneuverable and efficient way [18]. Since different active layer films prepared by diverse solvents have, respectively, special surface energy, reflecting the surface composition of blend film. Furthermore, the performance of BHJ solar cell in part depends on whether polymer or [6,6]-phenyl-C71-butyric acid methyl ester (PC_71_BM) would be enriched on the top surface of the blend film [19]. Besides, mixed solvents can also be effectively used to tune the miscibility of the donor and acceptor components, the domain size and the morphology of active layer, which play an important role in optimizing the photovoltaic performances for PSCs [20,21,22].

**Scheme 1:**
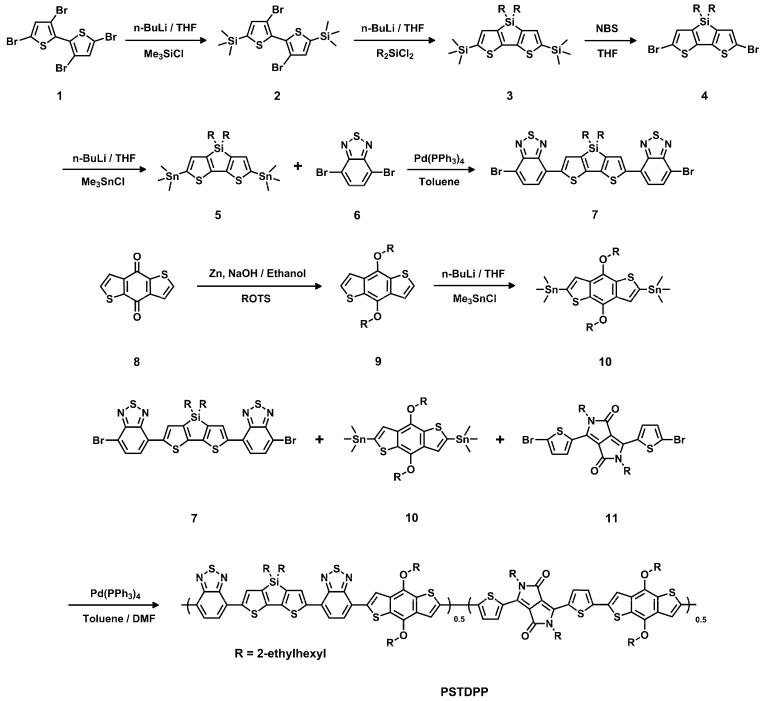
Synthesis route of the polymer.

In this study, we adopt an alternative approach for designing copolymer by “doping” a D–A fraction to another D–A copolymer by chemical synthesis. We present a new fully conjugated random copolymer, PSTDPP (see Scheme 1), containing two donor units, siloledithieno (SDT) [23] and benzodithiophene (BDT) [24], and two acceptors, benzothiadiazole (BT) [25] and diketopyrrolopyrrole (DPP) [26]. All of these units are frequently used building blocks for high efficient PSCs [27,28,29,30]. The copolymer was synthesized from them via Palladium-catalyzed Stille coupling reaction. The polymer synthesis concerns the copolymerization of three components, in which BDT acts as a bridge, linking the D–A fraction (BT-SDT-BT) and DPP units. As expected, the random polymer exhibits a broad absorption with a range of 500–800 nm and a proper HOMO energy level, which ensure its applicability for fabrication of PSC devices. The best PCE value of 4.38% was obtained by using mixed solvent of *O*-dichlorobenzene (ODCB) and chloroform (CF) with 1,8-octanedithiol (DIO) as additive, which is much better than those of devices using single CF or ODCB as processing solvent. The result suggests that using mixed solvent to prepare the active layer could improve the film characteristic effectively, leading to an enhanced PCE for PSCs.

## 2. Experimental Section

### 2.1. Materials and Synthesis

Solvents and other common reagents were obtained from Beijing Chemical Plant (Beijing, China). All other chemicals were purchased from commercial sources (Alfa Aesar (Heysham, UK), Acros (Geel, Belgium) and Sigma-Aldrich (Saint Louis, MO, USA)) and were used without further purification unless otherwise stated. Tetrahydrofuran, toluene, and chloroform were freshly distilled from appropriate drying agents prior to use. Compounds **1**, **2** [23], **8** [31], **10** [32] and **11** [33] were prepared according to the published literature. The detailed synthetic processes are illustrated in Scheme 1.

#### Compound **3**

Compound **2** and 40 mL THF were put into a flask, and cooled down to −78 °C by a liquid nitrogen–acetone bath. Then, 3.9 mL butyllithium solution in hexane (2.7 mol·L^−1^) was added dropwise for 5 min, and the reactant was stirred for another 15 min at that temperature. Subsequently, 1.95 g dichlorodi(2-ethyl-hexyl)silane (6 mmol) was added in one portion, and the cooling bath was removed and the reactant was stirred for 2 h under ambient temperature. Then, the reactant was poured into water and extracted by ethyl ether several times. After removal of volatiles, the residue was purified by silica gel chromatography using hexane as eluent and gave 2.26 g of compound **3** (yield 72%). The product was obtained as colorless oil. GC–MS: *m*/*z* = 562.2. ^1^H NMR (CDCl_3_, 400 MHz), δ (ppm): 7.06 (s, 2H), 1.71 (m, 2H), 1.51–1.14 (m, 16H), 0.91 (t, 6H), 0.85 (t, 6H), 0.76 (m, 4H), 0.31 (s, 18H).

#### Compound **4**

Compound **3** (1.69 g, 3 mmol) was dissolved into 20 mL THF, and *N*-bromosuccinimide (1.1 g, 6.17 mmol) was added in one portion. The reactant was stirred at ambient temperature for 4 h, and then extracted by diethyl ether. The volatiles were removed under vacuum, and the residue was purified by silica gel chromatography using hexane as eluent. The product was obtained as sticky colorless or pale yellow oil with a yield of 96%. MS (MALDI-TOF): *m*/*z* = 576.0. ^1^H NMR (CDCl_3_, 400 MHz), δ (ppm): 6.97 (s, 2H), 1.72 (m, 2H), 1.51–1.13 (m, 16H), 0.91 (t, 6H), 0.83 (t, 6H), 0.76 (m, 4H).

#### Compound **5**

Compound **4** (1.2 g, 2.51 mmol) and 20 mL ultra-dry THF were put into a flask. The clear solution was cooled down to −78 °C by a liquid nitrogen–acetone bath. Then, 2.4 mL butyllithium solution in hexane (2.3 mmol, 2.4 mol/L) was added dropwise. After stirring at −78 °C for 15 min, 7 mL trimethyltin chloride (1 mol/L) was added in one portion, and then the cooling bath was removed. After being stirred at ambient temperature for 2 h, the reactant was poured into cool water and extracted by diethyl ether several times. After removal of volatiles, the product (1.78 g, 2.39 mmol) was obtained as sticky pale green oil with a yield of 95% and used without any further purification. MS (MALDI-TOF): *m*/*z* = 744.1. ^1^H NMR (CDCl_3_, 400 MHz), δ (ppm): 7.03 (s, 2H), 1.68 (m, 2H), 1.4–1.13 (m, 16H), 0.90 (t, 6H), 0.83 (t, 6H), 0.74 (m, 4H).

#### Compound **7** [34]

Compound **5** (760 mg, 1.02 mmol), compound **6** (1.8 g, 6.11 mmol), Pd(PPh_3_)_4_ (0.100 g, 0.08 mmol), and toluene (15 mL) were put into a 50 mL flask and stirred under N_2_ protection for 20 min. The mixture was heated to 80 °C for 10 min, 100 °C for 10 min and 150 °C for 5 h. Upon cooling, the red residue was passed through a short silica plug eluting with CHCl_3_ (500 mL). All volatiles were removed *in vacuo* to give the crude product as a dark sticky solid. The material was then slurried in methanol (300 mL), filtered, loaded onto silica using CHCl_3_ and purified by flash chromatography using a hexanes/CHCl_3_ gradient. The product eluted as a red solution at 40% CHCl_3_. After fraction collection and solvent removal, the solid product was slurried in methanol (300 mL), sonicated for 30 min, and filtered. The solid was washed with copious amounts of methanol and acetone, and then dried under vacuum for 24 h. The product was collected as green/purple metallic colored powder. Yield = 45%. MS (MALDI-TOF): *m*/*z* = 844.0. ^1^H NMR (CDCl_3_, 400 MHz), δ (ppm): 8.17 (m, 2H), 7.82 (d, 2H), 7.68 (d, 2H), 1.54 (m, 2H), 1.33 (m, 4H), 1.28 (m, 4H), 1.21 (m, 8H), 1.10 (m, 4H), 0.82 (m, 12H).

#### PSTDPP

Compound **7** (0.42 g, 0.5 mmol), compound **10** (0.77 g, 1 mmol) and compound **11** (0.34 g, 0.5 mmol) were dissolved into anhydrous toluene (10 mL) in a three-neck flask. The solution was flushed with argon for 20 min, and then Pd(PPh_3_)_4_ (50 mg , 0.043 mmol) was added into the flask. The flask was subjected to three successive cycle of vacuum followed by refilling with argon. Then, the polymerization was carried out at 110 °C for 18 h under argon protection. After cooling to room temperature, the reaction mixture was dripped into 100 mL of methanol with vigorous stirring. The polymer precipitate was collected by filtration and transferred to a thimble. After drying, the crude polymer was subjected to Soxhlet extraction using methanol, acetone, hexane and chloroform as solvents. After final extraction with chloroform, the chloroform solution was concentrated to approximately 10 mL and then dripped into 100 mL of methanol with vigorous stirring. The polymer was collected by filtration and dried to afford a solid as the product. Yield = 72%, *M*_n_ = 21.6 kDa and PDI = 3.47.

### 2.2. Measurements

Mass Spectra were determined on Bruker microflex MALDI-TOF mass spectrometer (Bruker, Billerica, MA, USA). ^1^H NMR (400 MHz) was obtained on a Bruker avance 400 NMR Spectrometer (Bruker, Billerica, MA, USA) using tetramethylsilane as internal standard. UV–Vis absorption spectra were recorded on a Shimadzu UV-3600 UV–vis–NIR spectrophotometer (Shimadzu, Kyoto, Japan). The electrochemical cyclic voltammetry (CV) was conducted on an EG&G Princeton Applied Research VMP3 workstation (Bio-Logic, Claix, France) with Pt disk coated with the polymer film, Pt plate, and Ag/Ag^+^ electrode as working electrode, counter electrode and reference electrode respectively in a 0.1 mol·L^−1^ tetrabutylammoniumhexafluorophosphate (Bu_4_NPF_6_) acetonitrile solution. The current density–voltage (*J–V*) curves were obtained by a Keithley 2420 source-measure unit (Keithley, Cleveland, OH, USA). The photocurrent was measured under illumination using an Oriel Newport 150W solar simulator (AM 1.5G, Newport, Irvine, CA, USA), and the light intensity was calibrated with a Newport reference detector (Oriel PN 91150V, Newport, Irvine, CA, USA). The external quantum efficiency (EQE) measurements of the devices were performed in air with an Oriel Newport system (Model 66902, Newport, Irvine, CA, USA). The thickness of the active layer was measured on a Kla-TencorAlpha-StepD-120 Stylus Profiler (Kla-TencorAlpha-StepD-120 Stylus Profiler). X-ray diffraction (XRD) measurements were carried out in the reflection mode at room temperature using a 12-Kw D/max-rA X-ray diffraction system (12-kW D/max-rA X-ray diffraction system). The step size for all scans was 4 degree with a count time of 1 min per step. Atomic Force Microscope (AFM) images were obtained on a NanoscopeIIIa AFM Multimode Digital Instrument (Bruker, Billerica, MA, USA) in tapping mode.

### 2.3. Organic Field-Effect Transistor (OFET) Devices Fabrication and Measurement

OFETs were constructed on octadecyltrichlorosilane (OTS) modified SiO_2_/Si substrates with top-contact configuration. The 10 mg·mL^−1^ ODCB solutions of the polymers were spin-coated on the substrates in air at room temperature. The source and drain electrodes were patterned through the mask using thermal evaporation method. The channel length were 100 μm and the ratio of channel width to channel length *W*/*L* = 110. The mobility in the saturation regime was determined using the equation: *I*_DS_ = (μ_FET_·*W*·*C*_i_*/*2*L*)(*V*_G_ − *V*_T_)^2^, where *I*_DS_ is the drain-source current in the saturated regime, μ_FET_ is the field-effect mobility, *W* the channel width, *L* the channel length, *C*_i_ the capacitance SiO_2_ dielectric layer, *V*_G_ the gate voltage and *V*_T_ the threshold voltage.

### 2.4. PSC Devices Fabrication and Measurement

Polymer solar cell devices with the structure glass/indium tin oxide (ITO)/poly(3,4-ethylenedioxythiophene):poly(styrenesulfonate) (PEDOT:PSS)/polymer:PC_71_BM(*w*/*w*)/Ca/Al were fabricated as follows: The PEDOT:PSS solution was spin-coated on top of the cleaned ITO-coated glass substrate and the film thickness was approximately 40 nm. Then the coated substrates were transferred into a glove box. The polymer/PC_71_BM blend solution used in this study for spin-coating was 10 mg·mL^−1^ for the different solutions. The additive, DIO, was added prior to spin-coating process. The thickness of the active layer was controlled by changing the spin speed during the spin-coating process and distributed around 100 nm. The devices were completed by evaporating Ca/Al metal electrodes with an area of 4 mm^2^ as defined by masks. These layers were thermally evaporated at a pressure of 2 × 10^−6^ Torr. To optimize device performance, different D/A weight ratios (*w*/*w*) and different solution were used during the device fabrication process.

## 3. Results and Discussion

### 3.1. Optical and Electrochemical Properties

**Figure 1:**
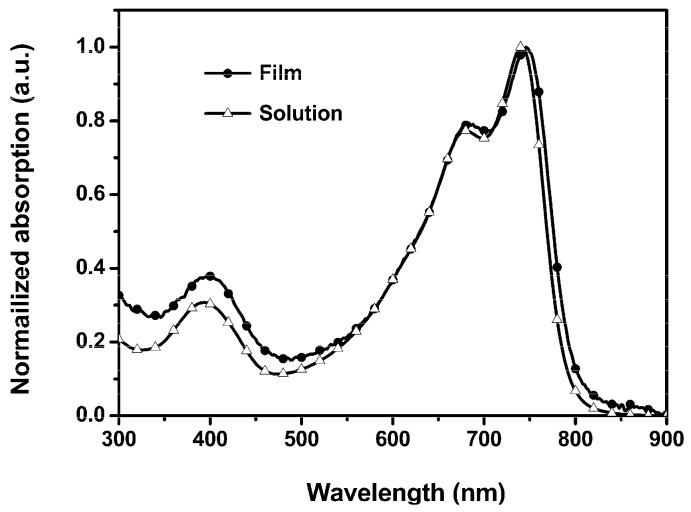
UV–vis absorption spectra of PSTDPP in 1 × 10^−5^ M chloroform solution and at solid state.

The optical property of PSTDPP was investigated by UV−vis absorption spectra both in chloroform solution and thin films. As shown in Figure 1, the polymer showed a relatively broad absorption in the UV–vis range. The absorption spectrum of PSTDPP in chloroform solution and at solid state showed absorption maximum at 677/738 and 683/747 nm, respectively. Compared with the absorption in solution, a very slight red shift of the spectrum for the solid state was observed. The optical band gap of PSTDPP can be calculated from the film absorption edge as 1.53 eV.

**Figure 2:**
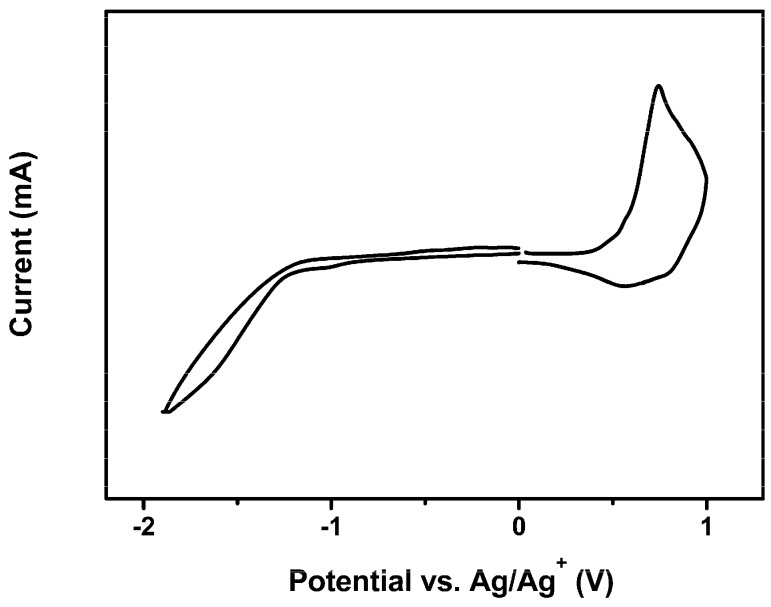
Cyclic voltammetry (CV) curves of PSTDPP at a scan speed of 50 mV s^−1^.

The electrochemical property of the polymer was investigated by cyclic voltammetry (CV) method. As shown in Figure 2, the polymer showed reversible *p*-doping/dedoping (oxidation/re-reduction) and n-doping/dedoping (reduction/reoxidation) processes. The energy levels of the highest occupied molecular orbital (HOMO) and the lowest unoccupied molecular orbital (LUMO) can be calculated from the onset oxidation and reduction potentials according to the equation: HOMO = –e (*E*^ox^ + 4.71) (eV) and LUMO = –e (*E*^red^ + 4.71) (eV) [35]. The electrochemical band gaps (*E*_g_^ec^) can be calculated from the equation: *E*_g_^ec^ = (*E*^ox^ –*E*^red^) (eV). The HOMO levels and the LUMO levels of PSTDPP were estimated to be at −5.15 eV and −3.47 eV, respectively. The corresponding electrochemical band gap of PSTDPP was 1.68 eV.

### 3.2. Crystallinity

**Figure 3:**
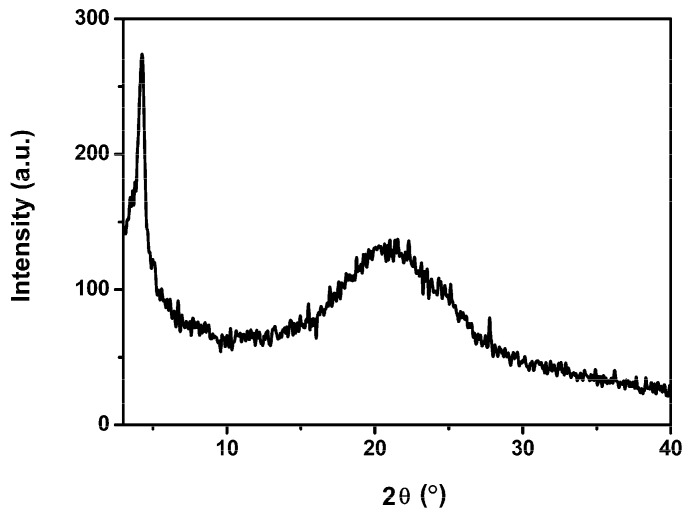
X-ray diffraction (XRD) pattern of PSTDPP film.

To gain a deeper insight of the crystallinity and molecular stacking mode of the polymer, X-ray diffraction (XRD) analysis of the polymer film spin-coated on the quartz substrate was operated (Figure 3). The PSTDPP film shows a distinct diffraction peak (100) at 2θ around 4.2°, indicating the chains form a interdigitation packing structure in the film with interchain *d*-spacing distance of 21.0 Å. The peak around 20°–25° for the polymer can be assigned to the face-to-face stacking of the aromatic groups arranged in the parallel direction [36]. The polymer exhibited good crystallinity and formed highly ordered structure in the film, which was expected to afford high hole mobility and facilitate charge carrier transport in BHJ solar cells.

### 3.3. Hole Mobility

**Figure 4:**
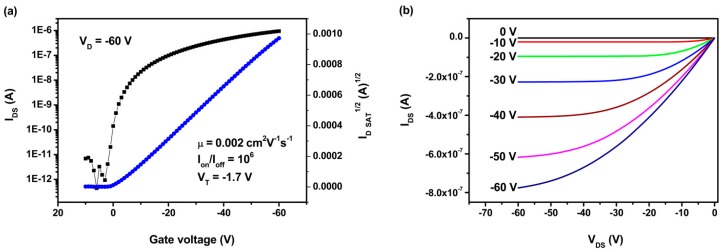
Transfer (**a**) and output (**b**) characteristics of PSTDPP.

The incorporation of siloledithieno and benzodithiophene donor units and strong electron-drawing benzothiadiazole and diketopyrrolopyrrole units into the polymer backbones was expected to enhance hole transport mobility of the polymers. The organic field-effect transistors (OFETs) with a conventional bottom-contact and bottom-gate configuration were fabricated using the polymer as the active layer. Figure 4 shows the representative transfer and output current–voltage plots for the OFETs fabricated on an OTS treated Si/SiO_2_ substrates. The devices showed a typical *p*-type transporting characteristic measured under ambient conditions. The PSTDPP based OFET exhibited relatively high hole mobility of 2.2 × 10^−3^ cm^2^·V^−1^·s^−1^ with an on/off ratio of 10^6^. The high mobility of the PSTDPP could be ascribed to the better molecular organization in the film. Furthermore, devices exhibited relatively high stability at ambient conditions. The devices were measured sequentially for about one month, and showed no obvious decline in the performance. The relatively high hole mobility is necessary for efficient charge transport in PSCs, which would be helpful to increase current density (*J*_sc_) and fill factors (*FF*) of the devices [37].

### 3.4. Photovoltaic Properties

Since the polymer possessed proper energy levels and a suitable high mobility, it could be expected to show excellent photovoltaic performance. PSCs were fabricated with a conventional sandwich structure of ITO/PEDOT:PSS/polymer:PC_71_BM/Ca/Al, where the active layer was composed of PSTDPP as the donor and PC_71_BM as the acceptor. Different D/A ratios (*w*/*w*) and processing solvents were systematically investigated to obtain the optimal device performance.

The device performances were firstly optimized by changing the D/A ratios of the polymer:PC_71_BM in ODCB solutions, and the optimal D/A ratio is 1:3 (Table 1). Then, further optimization was carried out by changing the processing solvents (from ODCB to CF or ODCB/CF blend). It was found that using CF as processing solvent was beneficial to the improvement of the open circuit voltage (*V*_oc_) and FF. Consequently, the solvent mixtures of ODCB and CF were chosen as processing solvents to investigate whether binary solvent can enhance the overall device performances. When using ODCB/CF (2/1, *v*/*v*) as processing solvent, a much better PCE of 3.79% was obtained with *J*_sc_ of 11.13 mA·cm^−2^, *V*_oc_ of 0.69 V and *FF* of 49.2%. At this point, all three parameters have been improved significantly compared with using ODCB as processing solvent. Finally, 1,8-diiodooctane (DIO) was added to the blend solution which was expected to tune the film morphology and device performance [38]. The resulting PCE for PSTDPP has grown to 4.38% by adding 2.5% DIO with further improvement of *J*_sc_/*FF* and minor loss of *V*_oc_.

**Figure 5:**
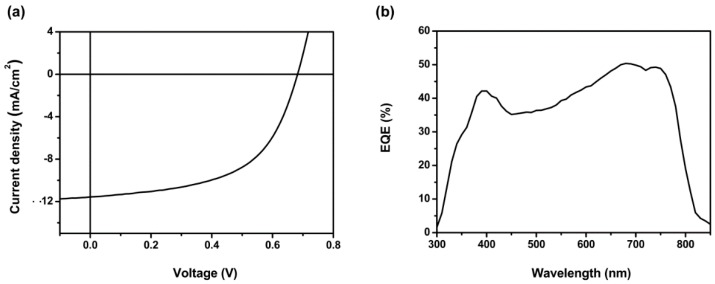
(**a**) *J–V* curve of PSTDPP based BHJ solar cells prepared under the optimal condition (D/A ratio = 1:3, using *O*-dichlorobenzene (ODCB) and chloroform (CF) (*v*/*v* = 2:1) with 2.5% DIO as processing solvent); and (**b**) EQE curve of the corresponding device.

The corresponding *V*_oc_, *J*_sc_, *FF* and PCE values of the devices are summarized in Table 1. Figure 5a shows the typical current density–voltage (*J–V*) curve of the PSC device fabricated the optimal condition by using the ternary solvents. The accuracy of the photovoltaic measurements for the optimal device can be further confirmed by the EQE of the devices as showed in Figure 5b. The EQE curve exhibited a broader photoresponse in the range of 390 to 750 nm with EQE values of 42%–50%. The *J*_sc_ calculated from integration of the EQE curve was 11.28 mA·cm^−2^, which shows around 2.4% mismatch compared with the *J*_sc_ value obtained from the *J–V* measurement.

**Table 1:** Photovoltaic properties of the PSTDPP/PC_71_BM-based bulk heterojunction (BHJ) solar cells fabricated with different solvents.

Solvent	D/A (*w*/*w*)	*V*_oc_ (V)	*J*_sc_ (mA cm^−2^)	*FF* (%)	PCE (%)
ODCB	1:1	0.51	5.53	34.8	0.98
ODCB	1:2	0.64	6.75	47.1	2.04
ODCB	1:3	0.63	9.89	37.9	2.35
ODCB	1:4	0.64	8.74	40.9	2.27
CF	1:3	0.69	1.68	50.9	0.59
ODCB:CF (2:1)	1:3	0.69	11.13	49.2	3.79
ODCB:CF (2:1) with 2.5%DIO	1:3	0.68	11.55	55.7	4.38

### 3.5. Water Contact Angle Characterizations

**Figure 6:**
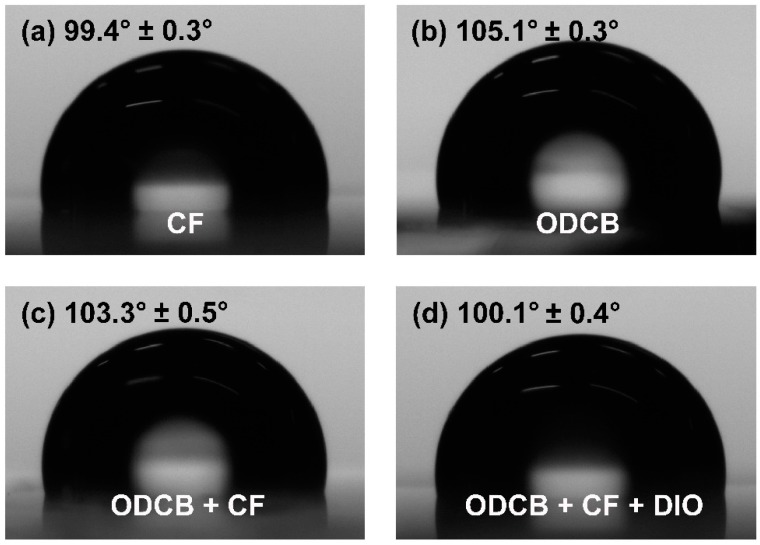
Water contact angle images of the top surface of (**a**) active-layer film prepared by CF; (**b**) active-layer film prepared by ODCB; (**c**) active-layer film prepared by ODCB:CF (2:1); and (**d**) active-layer film prepared by ODCB:CF (2:1) with 2.5% DIO.

Water contact angle characterizations were used to gain a deep insight of the surface components of the blend films processed by different solutions. As the decrease of water contact angle, the surface energy will increase and thus the top surface of the film will be more hydrophilic. Generally, water contact angle of polymer is bigger than that of PC_71_BM. For a blend film of BHJ solar cell composed by polymer and PC_71_BM, therefore, a smaller water contact angle indicates that PC_71_BM will be relatively enriched in the top surface of the blend film, and hence favorable ohmic contact with cathode will be formed, thus enhancing the photovoltaic performance of the corresponding device [19]. In fact, water contact angle of the blend film was affected, not only by the characters of polymer and PC_71_BM, but also by the preparation conditions of active layer. Figure 6 shows the respective water contact angles of four active-layer films prepared by different solvents. As shown, the water contact angle of the blend film prepared by CF is 99.4° ± 0.3°, which is far less than that of the blend film prepared by ODCB (105.1° ± 0.3°). Using the mixed solvent of ODCB/CF (2/1, *v*/*v*) to prepare the blend film could decrease the water contact angle and the corresponding value is 103.3° ± 0.5°. Interestingly, when adding some amount of DIO to the mixed solvent, a smaller water contact angle of 100.1° ± 0.4° was observed similar to the blend film prepared by CF, indicating that PC_71_BM was relatively enriched in the top surface of the blend film in both cases and the corresponding BHJ solar cells might represent relatively high photovoltaic performances accordingly [39,40]. Admittedly, this is just a possibility because the performance of PSC has a lot to do with the morphology of the active layer. Therefore, the analysis of morphology will be discussed in the following section.

### 3.6. Film Morphologies

**Figure 7:**
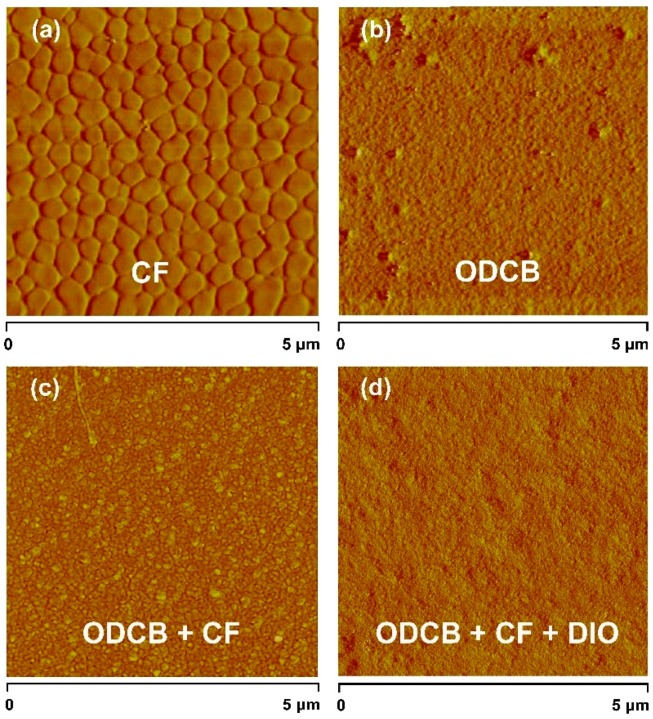
AFM phase image of PSTDPP/PC_71_BM blend film prepared by different solvents. (**a**) CF; (**b**) ODCB; (**c**) ODCB:CF (2:1); and (**d**) ODCB:CF (2:1) with 2.5% DIO. The size of the AFM image is 5 μm × 5 μm.

Atomic force microscopy (AFM) was used to characterize the morphology of the active layers spin-coated from different solutions. AFM phase image of the blend film prepared by CF (Figure 7a) reveals a high level of aggregation in the active layer and the surface roughness of the film is 0.819 nm. The very clear large domains with sizes of hundreds of nanometers would diminish exciton migration to the D–A interface and influence the following charge separation and collection [41], leading to the worst performance for PSTDPP based PSCs. Using ODCB as the preparation solvent, the morphology of active layer has improved somewhat and the surface roughness becomes a little smoother (root mean square (RMS) roughness = 0.768 nm), but there are still some unequally distributed granular aggregations on the surface of the blend film (Figure 7b). Consequently, the overall performance of the corresponding device showed only moderate improvement. When introducing the mix solvent of ODCB and CF to the active layer preparation, an obvious change of film morphology was seen in the Figure 7c. The granular aggregations have disappeared from the phase image and the domain size has reduced to tens of nanometers. Although the roughness of the film is 0.803 nm, the film morphology is much better than that of the above two cases, so the PCE exhibited a significant improvement. Finally, the optimized morphology was obtained by adding additive (DIO) to the mixed solvent. The roughness of the film has drop to 0.751 nm and the surface of the blend film becomes much smoother (Figure 7d), which is beneficial to the higher efficiency of exciton diffusion as well as charge separation [42]. In addition, combined with the water contact angle result, the best photovoltaic performance was eventually achieved for PSTDPP based PSCs with a ternary solvent.

## 4. Conclusions

In summary, a new random copolymer containing two donors (SDT and BDT) and two acceptors (BT and DPP), named as PSTDPP, has been synthesized and fully characterized. As expected, the random polymer exhibits a broad absorption with a range of 500–800 nm and a proper HOMO energy level, which ensure its applicability for fabrication of PSC devices. When using ODCB as processing solvent, a moderate PCE of 2.35% was obtained. To further optimize the device performances of PSTDPP/PC_71_BM-based PSCs, different processing solvents (from ODCB to CF or ODCB/CF blend) were systematically investigated. It was found that the solvent mixtures of ODCB and CF with small amount of DIO as additive could offer a much better active layer with PC_71_BM enriched surface composition and improved morphology, which led to a significantly enhanced PCE of 4.38% compared with using ODCB as processing solvent. These results indicate that constructing random copolymer with multiple donor and acceptor monomers could provide a new general method for designing photovoltaic polymers with optimized optical and electrochemical properties. In addition, introducing proper mixed solvents to the active layer preparation is a very promising approach to improve the characteristics of the film, which would be very conducive to achieving high PSC performance.
